# MetInfilt: A prospective trial highlighting the importance of the histological growth pattern in brain metastases

**DOI:** 10.1016/j.tranon.2025.102480

**Published:** 2025-07-24

**Authors:** Martin A. Proescholdt, Tommaso Araceli, Karl-Michael Schebesch, Christian Doenitz, Christina Wendl, Katja Evert, Ekaterina Noeva, Julius Hoehne, Markus J. Riemenschneider, Daniela Hirsch, Nils Ole Schmidt, Daniela Sparrer, Florian Lüke, Daniel Heudobler, Tobias Pukrop, Raquel Blazquez

**Affiliations:** a Department of Neurosurgery, University Hospital Regensburg, Regensburg, Germany; b Wilhelm-Sander Neuro-Oncology Unit, University Hospital Regensburg, Regensburg, Germany; c Department of Neurosurgery, Klinikum Nürnberg, Nürnberg, Germany; d Neuroradiology Branch, Department of Radiology, University Hospital Regensburg, Regensburg, Germany; e Institute of Pathology, University Regensburg, Regensburg, Germany; f Department of Neuropathology, University Hospital Regensburg, Regensburg, Germany; g Center for Translational Oncology (CTO), University Hospital Regensburg, Regensburg, Germany; h Bavarian Cancer Research Center (BZKF), Regensburg, Germany; i Department of Internal Medicine III, Hematology and Medical Oncology, University Hospital Regensburg, Regensburg, Germany; j Fraunhofer Institute for Toxicology and Experimental Medicine ITEM-R, Regensburg, Germany

**Keywords:** Brain metastasis, CONVIVO, HGP, meningeal metastasis, MetInfilt, MMPI, MRI

## Abstract

**Background:**

While the histological growth pattern (HGP) of liver metastases is frequently evaluated, the same attention is often absent for brain metastases despite evidence suggesting its prognostic significance. This oversight may stem from the lack of a standardized method for assessing the HGP at the macro-metastasis / brain parenchyma interface (MMPI_brain_). MetInfilt is the first prospective, imaging-guided trial aimed at standardizing the collection and analysis of the HGP at the MMPI_brain_.

**Methods:**

We recruited fifty patients. The MMPI_brain_ was identified using preoperative contrast-enhanced T1-weighted MRI. Intraoperative confocal microscopy (CONVIVO) visualized the MMPI_brain_, while a YELLOW 560 nm filter in the surgical microscope facilitated precise tissue sampling. Samples from the MMPI_brain_ and the core of the metastasis were collected for postoperative histological confirmation.

**Results:**

The protocol achieved successful tissue acquisition from the MMPI_brain_ in 93.2 % of patients, meeting the study's primary endpoint. Preoperative MRI patterns strongly correlated with infiltrative HGPs, and CONVIVO accurately visualized the MMPI_brain_ intraoperatively. Exploratory analyses suggest that infiltrative HGPs might negatively impact patient prognosis and represent a potential risk of meningeal metastasis.

**Conclusions:**

Our neurosurgical protocol allows the successful and precise acquisition of tissue from the MMPI_brain_ through presurgical imaging, intraoperative microscopy, and fluorescence-assisted sampling. The evaluation of the HGP in our limited patient cohort highlights its potential clinical significance and supports the urgent necessity to investigate it further for the benefit of patients with brain metastases.

**Clinical trial registration number:**

Z-2019–1307–9.

## Highlights

• The MetInfilt protocol is suitable for the standardized sample acquisition from the MMPI_brain_.
• Preoperative MRI strongly correlates with infiltrative HGPs.
• CONVIVO accurately visualized the MMPI_brain_ intraoperatively.
• Infiltrative HGPs might negatively impact patient prognosis and represent a potential risk of developing meningeal metastasis.

**List of abbreviations**
AI: Artificial intelligence
BrM: Brain metastasis
CE: Contrast enhancement
CI: Confidence Interval
CK: Cytokeratin
CNS: Central nervous system
CSF: Cerebrospinal fluid
Dx: Diagnosis
FFPE: Formalin-fixed paraffin-embedded
FL: Fluorescein sodium
GFAP: Glial fibrillary acidic protein
HGP: Histological growth pattern
HR: Hazard Ratio
ICR: Interquartile range
IHC: Immunohistochemistry
KPS: Karnofsky Performance Status
MeM: Meningeal metastasis
MITF-1: Microphthalmia-associated transcription factor 1
MMPI_brain_: Macro-metastasis / brain parenchyma interface
MMPI_liver_: Macro-metastasis / liver parenchyma interface
MRI: Magnetic resonance imaging
N/A: Not assessable
OS: Overall survival
PFS: Progression-free survival
PMD: Pachymeningeal disease
ROI: Region of interest

## Introduction

The histological growth pattern (HGP) is, besides the resection status (R 0–2), one of the current standard information included in the pathological reports of resected colorectal liver metastases. Due to its enormous prognostic value, the diagnostic determination of the HGP was recommended in the International Consensus Guidelines in 2017 for the first time [1]. Since then, many other studies have corroborated the clinical value of the HGPs in patients with liver metastases from various tumor types [2]. The main HGPs described for hepatic metastases are desmoplastic, pushing, or replacement [3,4]. The differences between these three categories rely on distinct morphological features observed at the interface between the tumor cells of the macro-metastasis and the surrounding liver parenchyma, also known as the macro-metastasis / liver parenchyma interface (MMPI_liver_). Liver metastases exhibiting desmoplastic or pushing HGPs are characterized by a clear separation between tumor cells and the surrounding liver parenchyma. In the desmoplastic HGP, this border is marked by a fibrotic rim enclosing the metastasis. In contrast, the pushing HGP lacks such a rim, with tumor cells compressing the adjacent parenchymal cells of the liver tissue. Additionally, desmoplastic metastases often show a dense immune cell infiltrate at the interface between the fibrous rim and liver parenchyma. In liver metastases with a replacement HGP, cancer cells infiltrate the liver parenchyma directly, coming into contact with and gradually replacing hepatocytes [5].

The HGPs in liver metastasis are associated with patient outcomes [6,7], recurrence [8] and therapy response [9]. In this context, the desmoplastic HGP is associated with a better prognosis, while the replacement HGP is often linked to therapy resistance and recurrence [5]. Due to its clinical relevance, the assessment of the HGP is part of the pathological diagnosis and guides clinical decisions in patients with hepatic metastases.

In contrast to the importance of HGPs in liver metastases, the HGP of resected brain metastases is neither considered in daily routine nor current guidelines, despite its potential prognostic significance [10]. These circumstances may be because surgical resection focuses on the contrast-enhancing tumor mass, and therefore, the neuropathologist does not routinely receive tissue from the macro-metastasis / brain parenchyma interface (MMPI_brain_)_._ In addition, the HGP is not uniform everywhere at the MMPI_brain_. From autopsy studies [11] and a previous prospective biopsy trial [10] we already know that brain metastases do not grow infiltrative at the entire circumference but may show only focal areas of infiltration. However, the region with the most aggressive HGP (infiltrative) determines the prognosis [10,12]. Despite our prior prospective biopsy trial conferred important information regarding the potential impact of the HGP at the MMPI_brain_ on patient prognosis [10], the main limitation was the lack of a targeted acquisition strategy of the MMPI_brain_ samples. Thus, a neurosurgical protocol that identifies this region preoperatively and obtains tissue samples from this area using an imaging-guided procedure and intraoperative neuronavigation was necessary to histologically examine this region in a reliable manner.

For the development of this targeted sampling strategy, we investigated, in the first step, magnetic resonance images (MRIs) of patients with brain metastases retrospectively [13]. This study identified four specific contrast enhancement (CE) patterns. Two MRI CE patterns displayed regularly shaped borders (“rim-enhancing” and “spherical”), and two showed an irregular delineation (“breakout” and “diffuse”), suggesting differences in the growth patterns of brain metastases. Interestingly, the breakout cohort's patient outcome was significantly worse than the other groups [13]. However, due to the retrospective evaluation of the MRIs, it was not possible to investigate the HGPs of the corresponding breakout regions histologically. Nevertheless, this observation was necessary for the MRI-guided specification of the targeted sampling strategy of the current trial.

Additionally, in a recent prospective neurosurgical trial [14] we demonstrated that the use of fluorescein sodium (FL) in combination with the YELLOW 560 nm filter as a method to visualize residual tumor tissue during brain metastasis resection provides a better postoperative result, which translates into better survival for brain metastasis patients.

Given the preoperative specification of the region of interest (ROI) with the most aggressive HGP for the targeted biopsy using MRI [13] and the visualizing possibilities of the metastatic cells at the MMPI_brain_ intraoperatively [[14], [15], [16]], we designed the current trial accordingly. In this prospective neurosurgical trial that we called MetInfilt (= Metastatic Infiltration), we combined the MRI-guided acquisition of biopsies with intraoperative confocal microscopy (CONVIVO) and FL-assisted resection. The primary endpoint of the MetInfilt trial was the feasibility of the neurosurgical protocol to acquire tissue from the MMPI_brain_ successfully. This would result in a standardized intraoperative approach for sampling the MMPI_brain_ and subsequent postoperative assessment of the HGP in brain metastases. Besides this primary endpoint of the trial, we investigated additional exploratory endpoints such as overall survival (OS), progression-free survival (PFS), and development of meningeal metastasis after resection of brain metastasis, to preliminarily examine the clinical importance of the HGP in our limited patient cohort.

## Results

### Imaging–guided biopsy of the macro-metastasis / brain parenchyma interface (MMPI_brain_)

**Fig. 1:**
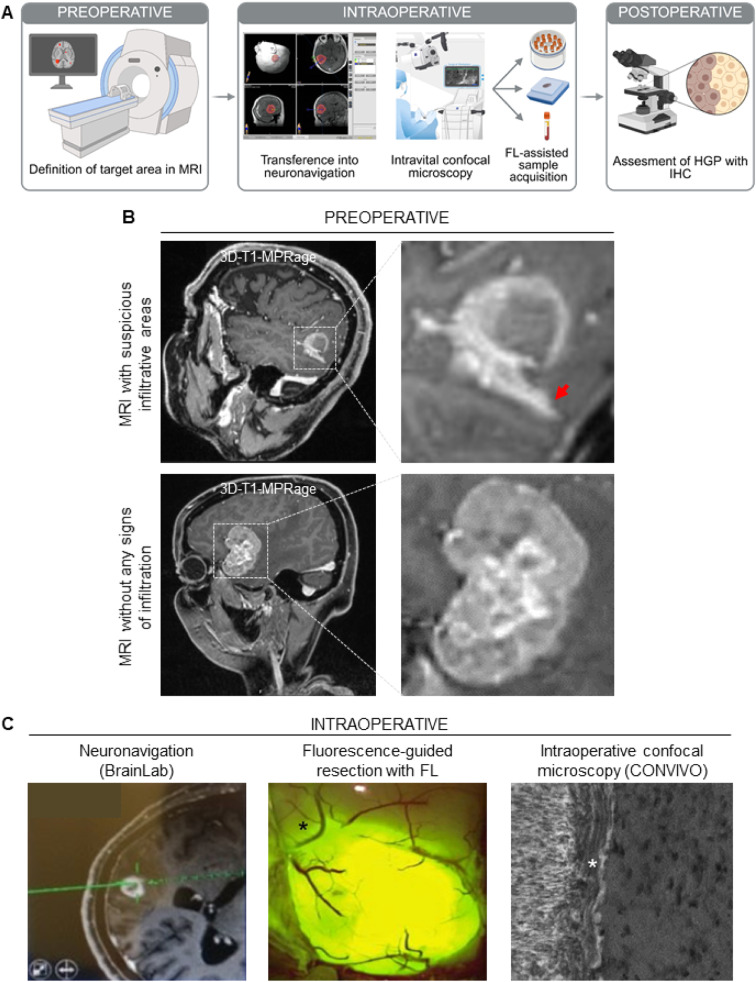
Neurosurgical protocol of the MetInfilt trial. (**A**) Schema depicting the workflow and sampling strategy of the trial. Image created with BioRender. (**B**) Preoperative assessment of the areas suspected of infiltrative growth was done by MRI. The red arrow indicates a potential infiltrative region. (**C**) Intraoperative strategy for sample acquisition. Left: Transfer of the MRI scans, including the target points, into the neuronavigation system to allow intraoperative detection of target areas. Middle: Fluorescein sodium (FL) application and use of the appropriate light filter to illustrate central and peripheral tumor areas. Right: Use of CONVIVO confocal laser endomicroscopy for live visualization of the MMPI. The asterisk indicates the region identified in the MRI.

The MetInfilt trial aimed at standardizing the tissue sampling of the MMPI_brain_. For this, we developed a dedicated neurosurgical protocol to enable the targeted acquisition of samples from the MMPI_brain_ based on presurgical MRI scans and supported by intraoperative confocal microscopy (CONVIVO) and FL-assisted resection (Fig. 1A). First, the study team members reviewed the presurgical MRI scans and determined the most relevant areas potentially representing tumor cell infiltration at the MMPI_brain_ (Fig. 1B). We visually analyzed the CE demarcation lines of the brain metastasis on a 3D-T1-MPRage sequence with special regard to sharpness using sagittal, coronal, and transversal planes, as previously published [13]. Areas of the metastasis with poorly defined, blurry CE demarcation lines were suspected to be infiltrative (Fig. 1B, top). Bright and well-defined CE demarcation lines of the metastasis were assumed to represent non-infiltrative regions (Fig. 1B, bottom). Subsequently, the MRI scans were transferred into the neuronavigation system (Brainlab Cranial, Germany) (Fig. 1C, left). Next, FL 10 % was applied for the intraoperative visualization of the metastasis and its border areas (Fig. 1C, middle), as previously described [14]. Confocal laser endomicroscopy (CONVIVO) was utilized to directly visualize the MMPI_brain_ intraoperatively (Fig. 1C, right) [15,16]. Biopsies were acquired from the selected areas (interface at the resection margin = MMPI_brain_ and core of the metastasis).

The median operative time in the MetInfilt study was 170.7 min (range 124.7 - 233.5 min), which was not significantly prolonged compared to patients receiving brain metastasis resection outside of the MetInfilt trial (median 165.7 min; range 123.4 - 201.33 min; *p* = 0.178). The study protocol did not induce additional surgical or neurological morbidity (*p* = 0.672 and 0.772, respectively).

### Histological confirmation of the HGP at the MMPI_brain_

**Fig. 2:**
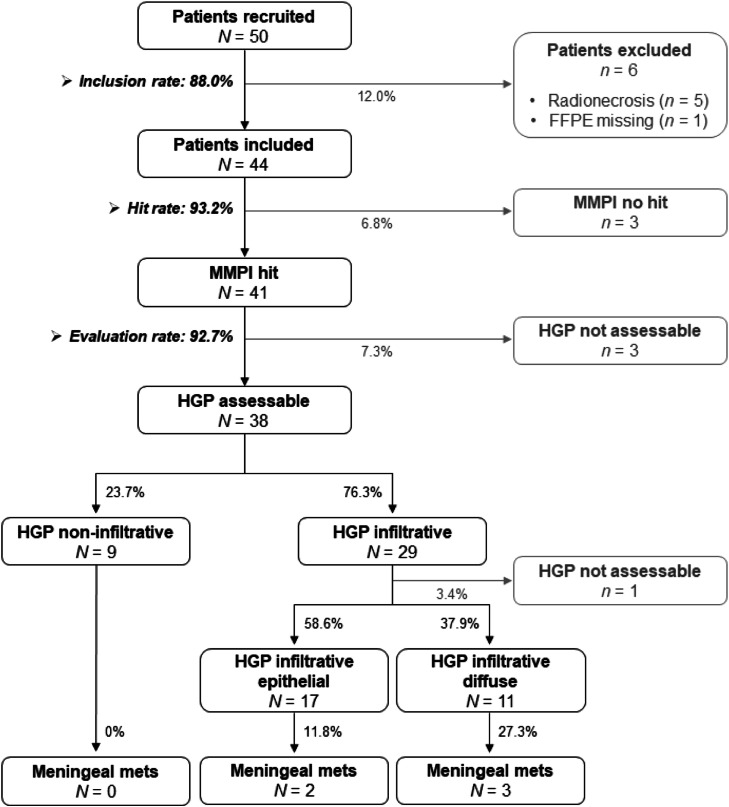
CONSORT statement of the MetInfilt trial. Flow diagram of the progress through the phases of the patient and sample inclusion and the postoperative histological analysis of the HGP in MMPI_brain_ samples.

The HGP at the MMPI_brain_ was investigated by histology and immunohistochemistry (IHC) of the formalin-fixed paraffin-embedded (FFPE) samples. Of the 50 patients recruited for the study, six patients (12 %) had to be excluded (Fig. 2, Supplementary Table 1). One patient was excluded because no FFPE samples could be acquired. The other five patients excluded presented with a contrast-enhancing, symptomatic, and progressive mass lesion, which caused the decision of the interdisciplinary neuro-oncologic board to resect it. However, the histological analysis only revealed posttherapeutic changes (radionecrosis) with reactive astrogliosis and macrocytic infiltration without any detectable tumor cells in either the interface or the core of the metastasis (Supplementary Figure 1). This observation was confirmed by the routine neuropathological diagnosis. Those cases had to be excluded from the analysis since there was no evidence of metastatic tumor cells in any of the acquired samples, and thus, the HGP at the MMPI_brain_ could not be determined. The primary tumor in these patients was lung cancer (*n* = 2), kidney cancer (*n* = 1), esophagus (*n* = 1), and malignant melanoma (*n* = 1). All patients received radiation treatment prior to metastatic resection (4 stereotactic fractionated radiation only; 1 whole brain radiation plus dosage boost within the metastatic lesion). In addition, all patients received systemic treatment (3 immune checkpoint inhibition, 2 targeted treatments, and 1 chemotherapy). These patients showed a trend to be older (67.8 vs. 59.7 years, *p* = 0.077); however, no significant difference was found in the primary tumor distribution (*p* = 0.239) or tumor volume (*p* = 0.331). Interestingly, the CONVIVO endomicroscopy findings of the potential MMPI were classified as inconclusive in all four patients where the CONVIVO images were available (Supplementary Fig. 1A, Supplementary Table 1). In these cases, no MMPI could be identified by endomicroscopy.

From the 44 patients that were included in the analysis, the MMPI_brain_ was successfully acquired in 93.2 % of the cases (41/44) (Fig. 2, Supplementary Table 1), meeting the primary endpoint of the study (hit rate >70 %). The successful acquisition of the MMPI_brain_ was defined as the presence of tumor cells within the gliosis (GFAP-positive area) in the MMPI_brain_ sample.

Next, we determined the HGP of the metastases at the MMPI_brain_. In 3/41 patients (7.3 %), the HGP could not be reliably assessed due to the limited brain tissue. Interestingly, the CONVIVO endomicroscopy findings of the potential MMPI_brain_ were classified as inconclusive in 2/3 of these patients (Supplementary Table 1).

**Fig. 3:**
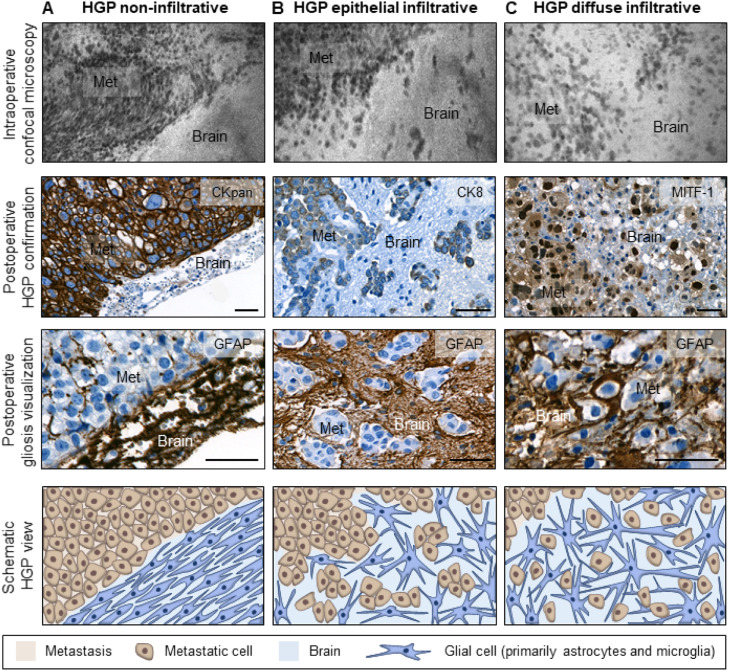
Evaluation of the HGP at the MMPI_brain_. Representative pictures of (**A**) non-infiltrative, (**B**) epithelial infiltrative, and (**C**) diffuse infiltrative HGPs visualized by intraoperative confocal microscopy and postoperative IHC. Tumor cells were stained using antibodies against pan-cytokeratin (CKpan), cytokeratin 8 (CK8) and microphthalmia-associated transcription factor 1 (MITF-1). The brain parenchyma (gliosis) was visualized using an anti-glial fibrillary acidic protein (GFAP) antibody. Scale bars represent 50 µm. Schematic pictures depicting the different HGPs are also shown. The non-infiltrative HGP shows a clear demarcation between the gliosis and the metastasis; the epithelial infiltrative HGP is characterized by groups of tumor cells inside the gliosis; and the diffuse infiltrative HGP displays single tumor cells mixed up with the gliosis.

We categorized the HGP of the remaining 38 patients (92.7 %) in which the HGP could be undoubtedly evaluated into three groups: non-infiltrative, epithelial infiltrative, and diffuse infiltrative, as previously published [4]. Metastases with non-infiltrative HGPs are characterized by a clear demarcation between the metastasis and the surrounding brain parenchyma (GFAP-positive areas). In contrast, infiltrative HGPs lack a clear separation between tumor and brain tissue, with tumor cells found interspersed within areas of gliosis. In the epithelial infiltrative pattern, tumor cells appear in strands, cell clusters, or glandular structures; whereas in the diffuse infiltrative pattern, they are more loosely arranged and infiltrate deeper into the adjacent brain tissue (Fig. 3). The key histopathological characteristics of the HGPs of brain metastases are summarized in Supplementary Table 2.

Nine of the 38 patients with an evaluable HGP (23.7 %) displayed a non-infiltrative HGP (Fig. 3A), whereas 29 (76.3 %) showed significant tumor cell infiltration into the adjacent brain. Of the patients with infiltrative HGPs, 17 (58.6 %) showed an epithelial growth pattern (Fig. 3B), and 11 (37.9 %) displayed a diffuse infiltrative HGP (Fig. 3C). One patient in the infiltrative group (3.4 %) could not be sub-categorized, as the morphology of the primary tumor (sarcoma) did not permit further classification (Fig. 2, Supplementary Table 1). Importantly, metastatic infiltration could already be visualized intraoperatively in the available CONVIVO images (Fig. 3 and Supplementary Table 1). In the metastases where a non-infiltrative HGP was determined histologically, the CONVIVO images revealed a clearly demarcated interface (Fig. 3A), whereas in metastases with epithelial and diffuse infiltrative HGPs, infiltrative metastatic cells could be detected in the gliosis already intraoperatively (Fig. 3B-C). Remarkably, in the CONVIVO images of diffuse infiltrative HGPs, the transition area between the metastatic cells and the brain was almost not distinguishable (Fig. 3C).

### Association between preoperative MRI and postoperative HGP confirmation by IHC

**Fig. 4:**
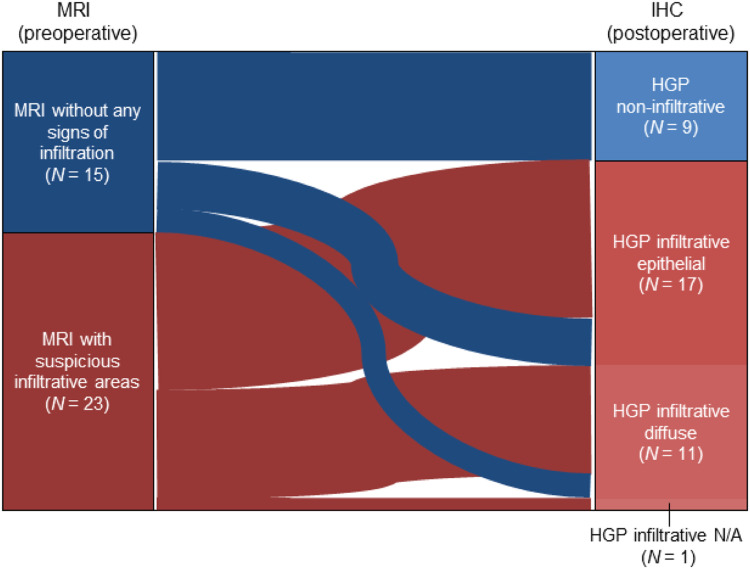
Correlation of preoperative MRI pattern and HGP confirmed postoperatively. Sankey chart showing the correlation between the CE pattern predicted by MRI preoperatively and the HGP determined by immunohistochemistry (IHC) postoperatively.

Following our previous publication [13] we analyzed preoperative MRI scans of the patients included in the MetInfilt trial and categorized them into four different CE patterns, two with delineated shaped borders (rim-enhancing and spherical) and two with patterns suggesting metastatic tumor infiltration (breakout and diffuse). Subsequently, we correlated the preoperative MRI findings with the results of the postoperative histological analysis of the HGP at the MMPI_brain_. Of the patients in which the HGP could be assessed (*n* = 38), 7 showed rim-enhancing (18.4 %), 8 showed spherical (21.1 %), 15 showed breakout (39.5 %), and 8 showed a diffuse (21.1 %) CE pattern (Supplementary Table 1). In all 23 cases in which the presurgical MRI scans suggested infiltration (breakout and diffuse), the postoperative histological analysis confirmed an infiltrative HGP at the MMPI_brain_. In contrast, in the 15 patients displaying MRI patterns without any signs of infiltration (rim-enhancing and spherical), we histologically confirmed tumor cell infiltration at the MMPI_brain_ in six cases (26.7 %) (Fig. 4, Supplementary Table 1). Thus, in our limited study population, MRI findings showed a specificity of 100 % (9/9 = zero false positives) and a sensitivity of 79.3 % (23/29 = six false negatives) for predicting infiltrative HGPs at the MMPI_brain_.

### Association between HGP and primary tumor

Next, we asked whether the primary tumor would define the HGP at the MMPI_brain_. For this, we evaluated the distribution of the HGPs among the most common tumors in our patient cohort (Supplementary Table 3). Lung cancer was the most common primary tumor (22/50, 44 %). From those patients with lung cancer brain metastasis in which the HGP could be assessed (*n* = 16, 72.7 %), 4 displayed a non-infiltrative HGP (25 %), 8 an epithelial infiltrative HGP (50 %), and 4 showed a diffuse infiltrative HGP (25 %) (Supplementary Table 1). The next most common primary tumor was malignant melanoma (7/50, 14 %). Of the 6/7 patients (85.7 %) in which the HGP was evaluated, one presented an epithelial infiltrative HGP (16.7 %), and 5 showed a diffuse infiltrative HGP (83.3 %) (Supplementary Table 1). The HGP of breast cancer patients (4/50, 8 %) was either epithelial infiltrative (*n* = 2, 50 %) or diffuse infiltrative (*n* = 2, 50 %) (Supplementary Table 1). All patients with colorectal cancer brain metastasis (4/50, 8 %), in which we could evaluate the HGP (3/4, 75 %), showed an epithelial infiltrative HGP (Supplementary Table 1). According to this data, we did not observe any statistically significant association between primary tumor and HGP (*p* = 0.312).

### Potential clinical implications associated with the HGP at the MMPI_brain_

**Fig. 5:**
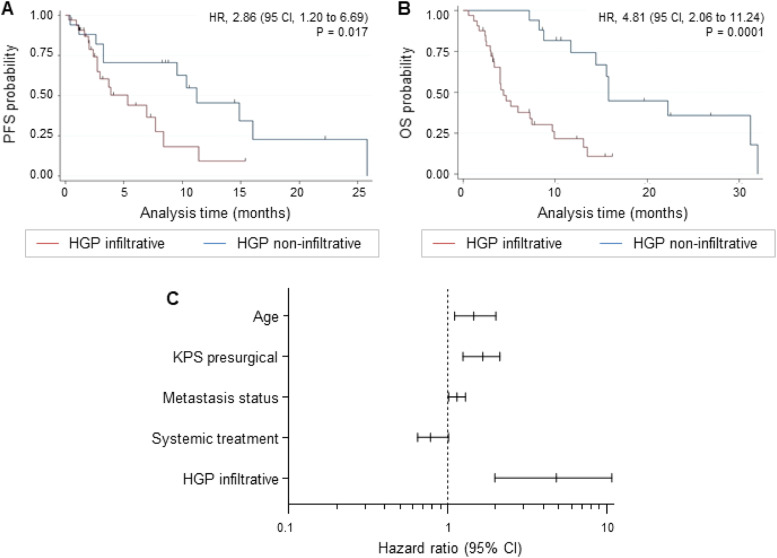
Impact of HGP on patient survival outcomes. (A-B) Kaplan-Meier curves showing (**A**) the progression-free survival (PFS) probability and (**B**) the overall survival (OS) probability stratified by infiltrative (red line) versus non-infiltrative HGP (blue line). Statistical analysis was performed by calculating log-rank analyses. (**C**) Forrest plot graphically summarizing the multivariate OS analysis results.

Following previous observations regarding the negative influence of metastatic infiltration on patient outcome [10,11], we evaluated the overall survival (OS) and progression-free survival (PFS) from the time point of metastasis resection of the patients included in the MetInfilt trial according to their HGP at the MMPI_brain_. It is important to emphasize that the limited size of our patient cohort restricts the analyses to an exploratory level. The median follow-up of the patients, in which the HGP could be assessed (*n* = 38), was 33 months (interquartile range (ICR) = 24.5). During the follow-up period, local recurrence occurred in 28 patients (56 %). Brain metastasis patients with an infiltrative HGP (*n* = 29) showed a significantly worse PFS (*p* = 0.017; HR = 2.86; Fig. 5A, Supplementary Table 4) and OS (*p* = 0.0001; HR = 4.81; Fig. 5B, Supplementary Table 4) compared with patients with a non-infiltrative HGP (*n* = 9). No statistically significant differences were found between brain metastases with infiltrative or non-infiltrative HGPs regarding tumor volume (*p* = 0.235), age (*p* = 0.176), presurgical Karnofsky Performance Status (KPS; *p* = 0.199), metastatic status (solitary, singular, multiple; *p* = 0.798), preoperative radiation (*p* = 0.759) or primary tumor (*p* = 0.470). Multivariate Cox regression analysis identified infiltrative HGPs as an independent prognostic factor for poor survival (Fig. 5C, Supplementary Table 5). Patients with epithelial (*n* = 17) and diffuse infiltrative HGPs (*n* = 11) showed no significant differences in PFS (*p* = 0.270; HR = 1.81 (95 CI, 0.628 to 5.258); Supplementary Figure 2A) or OS (*p* = 0.753; HR = 1.14 (95 CI, 0.480 to 2.750; Supplementary Figure 2B). Sample size calculations for PFS and OS are provided in the Supplementary Table 6. These data suggest that infiltrative HGPs may be associated with poorer survival outcomes compared to non-infiltrative patterns.

**Fig. 6:**
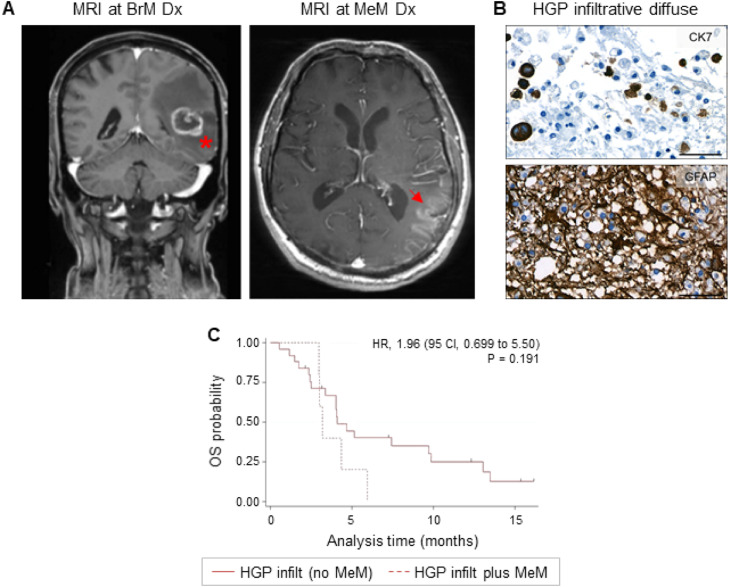
Occurrence of meningeal metastasis in patients with infiltrative HGPs. (A) MRI pictures showing a breakout CE pattern at the time of diagnosis (Dx) of brain metastasis (BrM) (left picture). The asterisk indicates the potential infiltrative area. Corresponding MRI picture at Dx of meningeal metastasis (MeM) (right picture) is shown. The red arrow indicates the site of meningeal metastasis. (**B**) IHC pictures depicting the diffuse HGP visualized by cytokeratin 7 (CK7, top) and the gliosis visualized by glial fibrillary acidic protein (GFAP, bottom). Scale bars represent 50 µm. (**C**) Kaplan-Meier curve showing the overall survival (OS) probability stratified by infiltrative HGP without meningeal metastasis (solid red line) versus infiltrative HGP with meningeal metastasis (dashed red line). Statistical analysis was performed by calculating log-rank analyses.

In a recent publication, we discussed the heterogeneous routes of metastatic dissemination that contribute to organ destruction and ultimately neurological death. In the brain parenchyma, secondary dissemination of metastatic cells—either by iatrogenic dissemination, direct extension (*per continuitatem*), or via the cerebrospinal fluid (CSF) space from existing brain metastasis—can lead to the development of meningeal metastasis [17]. Importantly, a significant correlation between the infiltration degree of brain metastasis and the risk of developing meningeal metastasis was previously reported by Dankner et al. [18]. Therefore, we examined the potential association between the HGP at the MMPI_brain_ and the occurrence of meningeal metastasis in our limited patient cohort. From the five patients (10 %) who developed meningeal metastasis during the follow-up period, all of them showed an infiltrative HGP (Fig. 2, Fig. 6A-B, Supplementary Table 1), suggesting a potential correlation between infiltrative growth patterns of brain metastases and a higher risk of meningeal dissemination. Patients with diffuse infiltrative HGPs (3/11, 27.3 %) seemed to be more prone to the development of meningeal metastasis compared with patients with epithelial infiltrative HGPs (2/17, 11.8 %) (Fig. 2, Supplementary Table 1). Moreover, patients with an infiltrative HGP that developed meningeal metastasis during the course of the disease showed a trend to have a shorter OS compared with patients with an infiltrative HGP but no meningeal spread (3.88 vs. 6.10 months; *p* = 0.191; HR = 1.96 (95 CI, 0.699 to 5.501)); however, no significant difference was found (Fig. 6C).

## Discussion

The primary endpoint of the MetInfilt trial consisted of the targeted acquisition of the MMPI in more than 70 % of the patients using the established imaging-guided neurosurgical protocol. This endpoint was successfully met since the MMPI area was hit in 93.2 % of the patients without increasing the surgical or neurological-related morbidity, demonstrating both feasibility and reproducibility of the protocol.

Our protocol included state-of-the-art technologies such as preoperative MRI, intraoperative confocal microscopy, and FL-guided resection to facilitate the accurate acquisition of samples from the MMPI_brain_. Thus, we asked to what extent the observations made by these technologies regarding the evaluation of the HGP matched with the histological results. Preoperative MRI scans predicted an infiltrative HGP at the MMPI_brain_ with a specificity of 100 % and a sensitivity of 79.3 %, meaning that all suspicious cases of infiltrative growth detected by MRI preoperatively were confirmed by histological analysis. Considering our data, we propose that MRI could be used to detect infiltrative cases specifically, as non-invasive methods to predict the HGP are extremely desirable. However, we recognize that evaluating the MRI scans by the human eye might have limitations. Indeed, 6/29 patients with infiltrative HGPs did not show any signs of infiltration on the preoperative MRI. This might be due to a human error in the assessment of the MRI pattern. Therefore, further efforts should be made to increase the accuracy of MRI as a non-invasive method to assess the HGP preoperatively. In this context, artificial intelligence (AI) algorithms could contribute to increasing the reliability of HGP recognition by MRI.

Moreover, we made use of FL to recognize the metastatic borders intraoperatively. FL has been shown to be useful in the surgical management of brain metastases and proven to be superior with regard to the volumetric extent of resection and overall outcome compared to white light resection [14]. Indeed, one study already demonstrated the successful tissue acquisition in the peripheral areas of the brain metastases using this method, but without focusing on the adjacent brain [19]. In the present study, FL was useful to identify the interface between the metastatic tissue and the neighboring brain parenchyma. Therefore, this method holds great promise for the targeted acquisition of the MMPI_brain_.

Further, we included intravital confocal microscopy in our protocol to visualize the MMPI_brain_ intraoperatively. CONVIVO pictures consistently reflected the HGPs confirmed postoperatively by IHC. Importantly, in most cases in which the MMPI could not be reliably assessed by postoperative histology, either because of the absence of metastatic tissue (radionecrosis) or the limited brain tissue, the CONVIVO endomicroscopy findings were classified as inconclusive. Thus, the MetInfilt trial highlights the feasibility of this method for the intraoperative visualization of the HGP. Confocal laser endomicroscopy has been reported to show high sensitivity and specificity in the detection of tumor tissue during brain tumor surgery, including brain metastases [20]. How much this novel technology will influence the intraoperative detection of resection margins in the context of infiltrative HGPs of brain metastases still needs to be evaluated [21]. Our observations indicate that detecting infiltrative tumor cells using CONVIVO may be helpful to guide supramarginal resection in brain metastases, leading to greater local control rates and potentially improved survival outcomes, as previously demonstrated [22]. Nevertheless, further studies specifically addressing this question are urgently needed to validate this observation.

The results of this study underscore the adecuacy of the neurosurgical protocol in enabling the targeted acquisition of the MMPI_brain_. In the patients included in this study, infiltrative HGPs were more prevalent than non-infiltrative patterns (76.3 % vs. 23.7 %, respectively). We observed a similar distribution in our previous study [10] although the difference there was less pronounced (64.1 % infiltrative vs. 35.9 % non-infiltrative). This discrepancy may be attributed to the untargeted nature of the earlier trial and suggests that the MetInfilt protocol provides a more suitable approach for the accurate assessment of HGPs at the MMPI_brain_. It is well established that growth patterns are not uniform along the tumor–brain interface; nevertheless, the presence of infiltrative growth—even in a limited region—is associated with poorer outcomes [1,10,12]. Therefore, precise acquisition of MMPI_brain_ samples and reliable HGP evaluation using the neurosurgical protocol implemented in the MetInfilt trial may offer clinical benefits for patients with brain metastases.

Importantly, the HGPs of brain metastases identified in this study partially mirror the well-characterized growth patterns observed in liver metastases. For instance, infiltrative HGPs in the brain infiltrate the surrounding parenchyma in a manner analogous to the replacement pattern described in hepatic metastases [5] though the mechanism of replacement growth in the brain needs to be further characterized. We further subclassified infiltrative growth into epithelial or diffuse types, based on the degree of cellular cohesion. Diffuse infiltrative cells exhibited low cohesion and an amoeboid morphology, reminiscent of the phenotype described by Friedl et al., which is associated with high plasticity and enhanced metastatic dissemination potential [23]. Our findings also suggest a potential link between diffuse infiltrative HGPs and an increased propensity for secondary dissemination and recolonization of the meninges [17]; however, these observations remain exploratory. In liver metastases, non-infiltrative HGPs are typically classified as either desmoplastic—characterized by a fibrotic rim separating the tumor from surrounding tissue—or pushing, where such a rim is absent [5]. Similarly, we observed the presence of a multilayered astrocytic rim encasing the lesion in a subset of brain metastases with non-infiltrative HGPs. Due to the limited number of non-infiltrative cases in our cohort (*n* = 9), we did not pursue further subclassification.

Even though our trial was not primarily designed to address further issues, we explored far beyond the feasibility of the protocol, and evaluated clinical aspects as purely explorative approaches to try to shed some light on whether the HGPs matter in brain metastases. First, we did not find any direct correlation between the HGP and the primary tumor of the corresponding metastasis. In our limited cohort, all melanoma and breast cancer patients showed infiltrative HGPs. In our previous trial [10] we also reported melanoma brain metastasis to be infiltrative rather than non-infiltrative. However, breast cancer patients also displayed non-infiltrative HGPs. Thus, besides this trend for the special case of melanoma metastases, the origin of the tumor does not seem to be directly correlated with the HGP of its metastasis in the brain. This contrasts with liver metastases, where tumor-type-dependent differences in the HGPs have been described [5]. Larger studies are needed to reliably investigate a possible correlation between the HGP and the tumor of origin in the context of brain metastases.

Despite the exploratory character of the analysis, the evaluation of the survival data revealed a strong association between infiltrative HGPs and a dismal patient prognosis. The impact of infiltrative HGPs on OS stands especially out in the multivariable analysis. Here, the HR of infiltrative HGP was 4.81, three times higher than the HR of other parameters that are usually used as a reference for patient prognosis, such as age (HR = 1.46), presurgical KPS (HR = 1.66), metastasis status (HR = 1.14) or systemic treatment (HR = 0.78). Presurgical radiation was also not associated with a worse OS, as previously postulated [24]. The specific type of infiltrative HGP (epithelial or diffuse) did not significantly influence survival. Thus, the preliminary results from our limited cohort regarding patient outcomes align with previous studies of brain [10] and liver metastasis [25].

In addition, our data suggest that infiltrative HGPs could be associated with the risk of secondary dissemination to the meninges, corroborating previous studies [18]. Though the MetInfilt trial was not specifically designed to evaluate this issue, it is noteworthy that all cases of meningeal metastasis occurred in patients with infiltrative HGPs. Secondary dissemination to the meninges is one of the several processes contributing to central nervous system (CNS) failure and neurological death [17]. Thus, a comprehensive understanding of the mechanisms underlying metastatic dissemination is essential for guiding future therapeutic strategies and monitoring approaches. In this context, the HGP holds great promise as a morphological indicator of secondary dissemination in brain metastasis [17] and thus a potential biomarker for predicting the development of pachymeningeal disease (PMD) following neurosurgery and radiation [26]. The implications of these processes, as well as the role of HGPs in organ failure and ultimately the cause of neurological death warrant further investigation.

## Conclusion

MetInfilt is the first prospective, imaging-guided trial aimed at standardizing the collection and analysis of the HGP at the MMPI_brain_. The neurosurgical protocol implemented in this study proved its feasibility and reproducibility for the targeted acquisition of the MMPI_brain_, addressing a significant gap in the field. Our results demonstrate the adecuacy of intravital CONVIVO microscopy in visualizing the MMPI_brain_ intraoperatively and point to MRI as a feasible non-invasive method to detect infiltrative HGPs at the MMPI_brain_. Moreover, our data suggest a potential association between infiltrative HGPs and adverse patient outcomes, including worse survival rates and an increased risk of secondary dissemination to the meninges. These preliminary insights might significantly impact the clinical management of brain metastases. However, given the limited sample size of our patient cohort and the fact that the study was not initially designed to address these specific questions, these findings should be considered exploratory and hypothesis-generating. Larger, multicenter studies are needed to validate the prognostic and predictive significance of HGPs in the context of brain metastasis. Moreover, molecular profiling of the distinct HGPs may help elucidate the underlying mechanisms driving pattern-specific metastatic colonization and organ destruction, and identify novel therapeutic targets to expand the currently limited treatment options for patients with brain metastases. We are confident that MetInfilt and the subsequent studies will pave the way for a potential new clinical standard, including the targeted tissue sampling during brain metastasis resection and pathological reporting of the HGP at the MMPI_brain_, similar to hepatic metastasis.

## Methods

### Patient information and inclusion criteria

We recruited 50 patients (20 female, 30 male, median age 62.2 years) undergoing microsurgical resection of a lesion suspected to be a brain metastasis between 2019 and 2023. Patients younger than 18 years of age, presenting with grade III eloquent tumor location [27] or with an inability to provide written informed consent were excluded. The primary endpoint of the MetInfilt trial was defined as the successful acquisition of MMPI_brain_ tissue confirmed by immunohistochemistry (IHC) in at least 70 % of patients. Most patients presented with brain metastases resulting from lung cancer (44 %), followed by melanoma (14 %), colorectal (8 %), and breast cancer (8 %). The clinical characteristics of the included patients are listed in Supplementary Table 3.

### Study approval

The study was approved by the ethics committee of the University Hospital Regensburg (protocol number: 19–1546–101). Prior to participation, written informed consent was obtained.

### Sex as a biological variable

Both females and males were involved in the study. Sex was not considered as a biological variable.

### MRI analysis

MR imaging of the included patients was performed according to a standardized scanning protocol; contrast-agent dosing was applied using a weight-adapted regimen. The different CE patterns of brain metastases on MRI were defined according to [13]. An MRI-based assignment of all brain metastases to one of the CE patterns was performed by two blinded readers (TA and MAP). In patients with multiple brain metastases, only the largest one in T1-post contrast was used for analysis. The CE patterns are reported in Supplementary Table 1.

### Evaluation of meningeal metastasis

Two investigators (TA and MAP) assessed the occurrence of meningeal metastasis in MRI images by analyzing the follow-up MRIs of all recruited patients. The results are reported in Supplementary Table 1.

### FL injection protocol

The FL injection protocol was performed as described previously [14]. Shortly, FL 10 % (ALCON, Germany) was applied following induction of general anesthesia in a dosage of 5 mg per kilogram bodyweight via a central venous line approximately 30–45 min before skin incision. For intraoperative visualization of the tumor and its border areas, a YELLOW 560 nm filter integrated into the surgical microscope (Carl Zeiss Meditec, Oberkochen, Germany) was used.

### Confocal laser endomicroscopy (CONVIVO)

CONVIVO (Carl Zeiss Meditec, Oberkochen, Germany) was utilized to directly visualize the MMPI_brain_ intraoperatively, as described [15,16]. The presence of metastatic infiltration at the MMPI_brain_ was assessed in the CONVIVO images by two blinded investigators (TA and MAP). The results are reported in Supplementary Table 1.

### Sample collection

Two to four wedge-shaped biopsies with a size of at least 3 × 3 mm were acquired from the selected areas (interface at the resection margin = MMPI_brain_ and core of the metastasis). Half of the samples were fixed in formalin and embedded in paraffin (FFPE) for histological analysis of the biopsied tissue. The remaining samples were snap-frozen in precooled isopentane and stored in a −80-degree freezer for further analysis. We developed a standard operational procedure with the research staff in the operation room at the time of durotomy to ensure rapid transfer of the biopsied tissue into the lab. The median time between tissue retrieval and sample freezing was 26 min (range 16 – 38 min).

### Histology and immunohistochemistry (IHC)

FFPE samples were sectioned (3 µm), deparaffinized, and stained with haematoxylin-and-eosin (H&E) or pretreated for IHC using standard techniques. An anti-glial fibrillary acidic protein (GFAP) antibody (DAKO Cytomation, Clone 6F2, Code No M0761, 1:2000) was used to detect the bordering brain within the MMPI_brain_; further, adjacent sections were stained using antibodies to label tumor cells. The IHC markers used according to the respective tumor of origin are listed in Supplementary Table 7.

### Assessment of the histological growth patterns (HGPs) of brain metastases

The HGP of brain metastases was assessed using light microscopy on high-quality H&E and IHC-stained sections from FFPE resection specimens of the MMPI_brain_ showing tumor cells and brain tissue (GFAP-positive area). The central region of the metastasis was not considered in the classification of the growth pattern. Since MMPI_brain_ specimens often exhibit heterogeneity in HGPs, the presence of even a small region showing infiltrative growth in the stained sections was sufficient to classify the patient into the corresponding HGP category.

The assessment was performed independently and in a blinded manner by four trained investigators (RB, MJR, MAP, KE), in accordance with previously published consensus criteria [4] as outlined in Supplementary Table 2. Consistency between observers was assessed by Cohen's Kappa statistic (*p* = 0.841).

### Statistical analysis

The sample size for the MetInfilt trial was calculated, resulting in a planned study population of fifty patients (Stata 16.1, Stata Corporation, College Station, TX, USA; Stata (RRID:SCR_012763)). Continuous variables were described as medians in addition to the ranges; categorical variables were reported as rates and proportions. Comparative analyses were executed by calculating non-parametric group testing for continuous variables and chi-square testing for categorical variables and their distribution. PFS and OS results were determined by the application of the Kaplan-Meier estimator. Factors influencing survival were evaluated as univariate analyses by calculating log-rank tests. Multivariate analysis of independent prognostic parameters was performed using Cox proportional hazards modeling. A p-value of <0.05 was considered statistically significant. Analyses were performed with Stata/IC (version 16.1, Stata Corp. College Station, USA).

## CRediT authorship contribution statement

**Martin A. Proescholdt:** Writing – review & editing, Writing – original draft, Visualization, Resources, Formal analysis, Data curation, Conceptualization. **Tommaso Araceli:** Writing – review & editing, Investigation. **Karl-Michael Schebesch:** Writing – review & editing, Investigation. **Christian Doenitz:** Writing – review & editing, Investigation. **Christina Wendl:** Writing – review & editing, Investigation. **Katja Evert:** Writing – review & editing, Resources, Investigation. **Ekaterina Noeva:** Writing – review & editing, Investigation. **Julius Hoehne:** Writing – review & editing, Investigation. **Markus J. Riemenschneider:** Writing – review & editing, Investigation. **Daniela Hirsch:** Writing – review & editing, Investigation. **Nils Ole Schmidt:** Writing – review & editing, Resources. **Daniela Sparrer:** Visualization, Writing – review & editing. **Florian Lüke:** Writing – review & editing. **Daniel Heudobler:** Writing – review & editing. **Tobias Pukrop:** Writing – review & editing, Conceptualization. **Raquel Blazquez:** Writing – review & editing, Writing – original draft, Visualization, Supervision, Project administration, Formal analysis, Data curation, Conceptualization.

## Declaration of competing interest

The authors declare that they have no known competing financial interests or personal relationships that could have appeared to influence the work reported in this paper.

## Data Availability

Anonymized data are available upon request for replication purposes. Inquiries should be made at datenspende@ukr.de.

## Appendix Supplementary materials

Image, application 1

Image, application 2

Image, application 3
