# Supplementary material for: MetInfilt: A prospective trial highlighting the importance of the histological growth pattern in brain metastases
Source: Transl Oncol. 2025 Jul 24;60:102480. doi: 10.1016/j.tranon.2025.102480 (PMC12311954; doi:10.1016/j.tranon.2025.102480)
Supplement: Supplementary file 1 [file mmc1.docx]

**Proescholdt et al._Supplementary Data**

Supplementary Figures

*
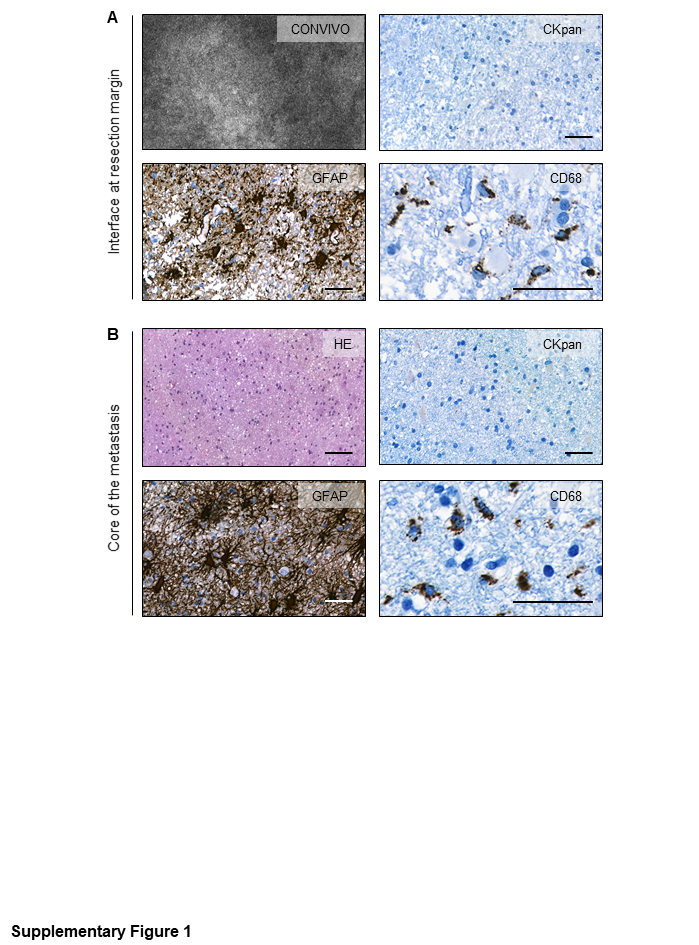
*

**Supplementary Figure 1: Visualization of radionecrotic tissue.** (**A-B**) Representative CONVIVO, HE and IHC pictures showing the absence of tumor cells (CKpan), the reactive astrocytosis (GFAP) and macrocytic infiltration (CD68) at (**A**) the interface at the resection margin and (**B**) the core of the metastasis as a consequence of radionecrosis. Scale bars represent 50 µm.

*
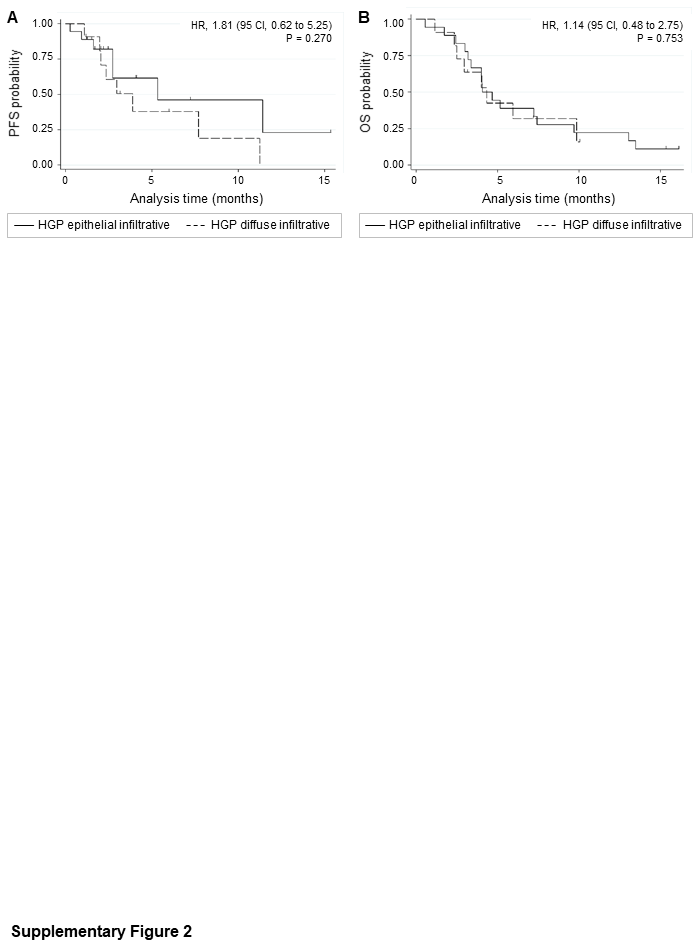
*

**Supplementary Figure 2: Impact of epithelial and diffuse infiltrative HGPs on patient survival.** Kaplan–Meier curves showing (**A**) the progression-free survival (PFS) probability and (**B**) the overall survival (OS) probability stratified by epithelial infiltrative (solid line) versus diffuse infiltrative HGP (dashed line). Statistical analysis was performed by calculating log-rank analyses.

Supplementary Tables

**Supplementary Table 1:** Patient data summary.

| **Pat#** | **Primary tumor** | **Included no (0), yes (1)** | **Exclusion reason** | **MRI (pre-OP)** | | **CONVIVO (intra-OP)** | **IHC (post-OP)** | | **Meningeal metastasis no (0), yes (1)** |
| --- | --- | --- | --- | --- | --- | --- | --- | --- | --- |
|  |  |  |  | **MRI pattern non-inf (0), inf (1)** | **MRI pattern (rim-enhancing, spherical, breakout, diffuse)** | **CONVIVO non-inf (0), inf (1), inconclusive (3)** | **HGP pattern non-inf (0), inf (1), no hit (3)** | **HGP pattern (non-inf, epithelial inf, diffuse inf)** |  |
| 1 | Lung | 1 |  | 1 | Diffuse | 1 | 1 | Diffuse | 0 |
| 2 | Lung | 1 |  | 0 | Spherical | 0 | 0 | Non-inf | 0 |
| 3 | Lung | 1 |  | 1 | Diffuse | 1 | 1 | Epithelial | 0 |
| 4 | Melanoma | 1 |  | 1 | Breakout | N/A | 1 | Diffuse | 0 |
| 5 | Head neck | 1 |  | 0 | Spherical | 1 | 3 |  | 0 |
| 6 | Lung | 1 |  | 0 | Rim | 1 | 1 | Epithelial | 0 |
| 7 | Breast | 1 |  | 1 | Breakout | N/A | 1 | Diffuse | 0 |
| 8 | Breast | 1 |  | 1 | Breakout | 1 | 1 | Diffuse | 0 |
| 9 | Melanoma | 1 |  | 1 | Breakout | 1 | 1 | Diffuse | 1 |
| 10 | Lung | 1 |  | 1 | Diffuse | N/A | 1 | Diffuse | 0 |
| 11 | Kidney | 1 |  | 1 | Breakout | 3 | N/A |  | 0 |
| 12 | Lung | 1 |  | 0 | Rim | 0 | 0 | Non-inf | 0 |
| 13 | Melanoma | 0 | Met-free | 1 | Breakout | N/A |  |  | 0 |
| 14 | Melanoma | 1 |  | 1 | Breakout | N/A | 1 | Diffuse | 0 |
| 15 | Melanoma | 1 |  | 1 | Breakout | 1 | 1 | Diffuse | 0 |
| 16 | Germ cell | 1 |  | 0 | Rim | 0 | 0 | Non-inf | 0 |
| 17 | Melanoma | 1 |  | 1 | Breakout | 1 | 1 | Diffuse | 1 |
| 18 | Melanoma | 1 |  | 1 | Breakout | 1 | 1 | Epithelial | 0 |
| 19 | Colorectal | 1 |  | 1 | Diffuse | 1 | 1 | Epithelial | 0 |
| 20 | CUP | 1 |  | 0 | Spherical | N/A | 0 | Non-inf | 0 |
| 21 | Colorectal | 1 |  | 1 | Breakout | 1 | 1 | Epithelial | 0 |
| 22 | Prostata | 1 |  | 0 | Spherical | 0 | 0 | Non-inf | 0 |
| 23 | Lung | 1 |  | 1 | Breakout | 1 | 3 |  | 0 |
| 24 | Lung | 1 |  | 0 | Spherical | N/A | 1 | Epithelial | 0 |
| 25 | Esophagus | 0 | Met-free | 1 | Breakout | 3 |  |  | 0 |
| 26 | Breast | 1 |  | 1 | Diffuse | N/A | 1 | Epithelial | 0 |
| 27 | Lung | 1 |  | 1 | Diffuse | 0 | 3 |  | 0 |
| 28 | Lung | 0 | Met-free | 0 | Rim | 3 |  |  | 0 |
| 29 | Lung | 1 |  | 1 | Breakout | 1 | 1 | Epithelial | 1 |
| 30 | Germ cell | 1 |  | 1 | Breakout | 1 | 1 | Epithelial | 0 |
| 31 | Lung | 1 |  | 0 | Spherical | 1 | N/A |  | 0 |
| 32 | Lung | 0 | No FFPE | 0 | Rim | N/A |  |  | 0 |
| 33 | Esophagus | 1 |  | 1 | Breakout | 1 | 1 | Epithelial | 0 |
| 34 | Lung | 1 |  | 1 | Breakout | 1 | 1 | Epithelial | 0 |
| 35 | Lung | 1 |  | 0 | Spherical | 0 | 0 | Non-inf | 0 |
| 36 | Lung | 1 |  | 0 | Rim | 1 | 1 | Diffuse | 1 |
| 37 | Sarcoma | 1 |  | 1 | Breakout | 1 | 1 | N/A | 0 |
| 38 | Esophagus | 1 |  | 0 | Spherical | N/A | 0 | Non-inf | 0 |
| 39 | Lung | 0 | Met-free | 0 | Rim | 3 |  |  | 0 |
| 40 | Lung | 1 |  | 1 | Diffuse | 1 | 1 | Epithelial | 0 |
| 41 | Colorectal | 1 |  | 0 | Spherical | 3 | N/A |  | 0 |
| 42 | Lung | 1 |  | 1 | Breakout | 1 | 1 | Epithelial | 0 |
| 43 | Lung | 1 |  | 0 | Rim | 1 | 1 | Diffuse | 0 |
| 44 | Cervix | 1 |  | 0 | Rim | 0 | 0 | Non-inf | 0 |
| 45 | Sarcoma | 1 |  | 1 | Diffuse | 1 | 1 | Epithelial | 1 |
| 46 | Breast | 1 |  | 1 | Diffuse | N/A | 1 | Epithelial | 0 |
| 47 | Lung | 1 |  | 0 | Rim | 0 | 0 | Non-inf | 0 |
| 48 | Lung | 1 |  | 0 | Rim | 1 | 1 | Epithelial | 0 |
| 49 | Colorectal | 1 |  | 0 | Spherical | 1 | 1 | Epithelial | 0 |
| 50 | Kidney | 0 | Met-free | 0 | Rim | 3 |  |  | 0 |

N/A = not assessable

**Supplementary Table 2:** Key histopathological characteristics of the growth patterns of brain metastases.

|  | **Non-infiltrative** | **Epithelial infiltrative** | **Diffuse infiltrative** |
| --- | --- | --- | --- |
| General architecture | Metastatic cells displace the adjacent brain tissue | Metastatic cells infiltrate the adjacent tissue as cell clusters, strands or glands | Metastatic cells infiltrate the adjacent tissue as single cells or small cell cohorts |
| Growth mechanism | Displacement +/-Compression | Infiltration | Infiltration |
| Borders | Sharp demarcated | Irregular | Irregular and no longer distinguishable |
| Tumor cell infiltration into brain parenchyma | - | + | + |
| Infiltration depth | n.a. | + | ++ |
| Astrogliosis reaction | +/- | + | ++ |
| Astrogliosis type | Localized, aligned | Widespread | Widespread |
| Astrocytic rim | +/- | - | - |
| Presence of tumor cells within the gliosis | - | + | + |
|  | Figure 3 A | Figure 3 B | Figure 3 C |

**Supplementary Table 3:** Clinical characteristics of the patient cohort and distribution of primary tumors.

| **Parameter** |  | **Entire population**  **(n; %)** |
| --- | --- | --- |
| **N** |  | 50 |
| **Age (median; range)** |  | 62.2 (28.5-81.7) |
| **Sex (f/m)** | f | 20 (40%) |
|  | m | 30 (60%) |
| **Metastasis status** | solitary | 2 (4%) |
|  | singular | 13 (26%) |
|  | multiple | 35 (70%) |
| **Metastasis timing** | synchronous | 18 (36%) |
|  | metachronous | 32 (64%) |
| **Preoperative KPS (median; range)** |  | 80 (50-100) |
| **Primary tumor** | lung | 22 (44%) |
|  | melanoma | 7 (14%) |
|  | breast | 4 (8%) |
|  | colorectal | 4 (8%) |
|  | esophagus | 3 (6%) |
|  | kidney | 2 (4%) |
|  | sarcoma | 2 (4%) |
|  | cervix | 2 (4%) |
|  | CUP | 1 (2%) |
|  | other | 3 (6%) |

**Supplementary Table 4:** Univariate analysis of factors associated with OS and PFS in the entire cohort.

| **Univariate analysis OS** |  |
| --- | --- |
| **Parameter** | **p-value** |
| Age | 0.023 |
| KPS presurgical | 0.012 |
| Metastasis status | 0.015 |
| Systemic treatment | 0.01 |
| HGP infiltrative | 0.0001 |
|  |  |
| **Univariate analysis PFS** |  |
| **Parameter** | **p-value** |
| Age | 0.008 |
| KPS presurgical | 0.204 |
| Metastasis status | 0.326 |
| Systemic treatment | 0.046 |
| HGP infiltrative | 0.017 |

**Supplementary Table 5:** Multivariate analysis of independent factors associated with OS and PFS.

| **Multivariate analysis OS** | |  |  |  |
| --- | --- | --- | --- | --- |
| **Parameter** | **HR** | **95% CI** | | **p – value** |
| Age | 1.456 | 1.105 | 2.002 | 0.007 |
| KPS presurgical | 1.658 | 2.125 | 1.245 | 0.001 |
| Metastasis status | 1.142 | 1.009 | 1.291 | 0.034 |
| Systemic treatment | 0.778 | 0.646 | 1.006 | 0.001 |
| Infiltrative HGP | 4.812 | 1.976 | 10.757 | 0.001 |
|  |  |  |  |  |
|  |  |  |  |  |
| **Multivariate analysis PFS** | |  |  |  |
| **Parameter** | **HR** | **95% CI** | | **p – value** |
| Age | 1.556 | 1.325 | 2.225 | 0.001 |
| KPS presurgical | 1.039 | 0.995 | 1.085 | 0.08 |
| Metastasis status | 3.017 | 1.042 | 9.869 | 0.034 |
| Systemic treatment | 0.667 | 0.454 | 1.106 | 0.042 |
| Infiltrative HGP | 2.862 | 1.12 | 6.691 | 0.03 |

**Supplementary Table 6:** Sample size calculation for survival analyses.

| **Analysis** | **Alpha** | **Power** | **Delta** | **HR** | **Estimated number**  **of events** |
| --- | --- | --- | --- | --- | --- |
| Progression – free survival | 0.050 | 0.800 | 1.0508 | 2.86 | 29 |
| Overall survival | 0.050 | 0.800 | 1.570 | 4.81 | 13 |

**Supplementary Table 7:** List of antibodies used in IHC staining for the different primary tumors.

| **Primary tumor** | **Antibody** | **Dompany** | **Dilution** |
| --- | --- | --- | --- |
| Lung cancer | CK5/6 | Dako | 1:200 |
|  | CK7 | Dako | 1:400 |
|  | CK8 | Diagnostic BioSystems | 1:100 |
|  | CK20 | Dako | 1:250 |
|  | P40 | Biocare Medical | 1:100 |
| Melanoma | MITF-1 | Cell Marque | 1:100 |
|  | HMB45 | Dako | 1:100 |
|  | MelanA | Leica | 1:50 |
| Colon | CK8 | Diagnostic BioSystems | 1:100 |
| Breast | CK7 | Dako | 1:400 |
|  | CK8 | Diagnostic BioSystems | 1:100 |
| Esophagus | CK7 | Dako | 1:400 |
|  | CK8 | Diagnostic BioSystems | 1:100 |
| Kidney | CKpan | Dako | 1:200 |
| Sarcoma | ActinASM | Diagnostic BioSystems | 1:500 |
|  | SATB2 | Cell Marque | 1:50 |
