# Supplementary figures and images for: MetInfilt: A prospective trial highlighting the importance of the histological growth pattern in brain metastases
Source: Transl Oncol. 2025 Jul 24;60:102480. doi: 10.1016/j.tranon.2025.102480 (PMC12311954; doi:10.1016/j.tranon.2025.102480)

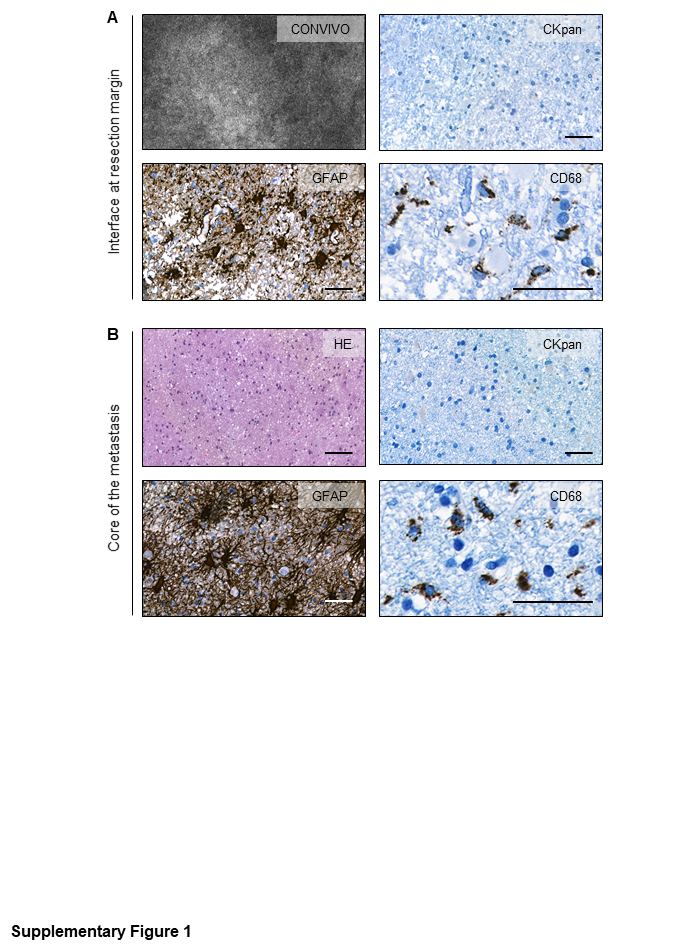

Supplement: Supplementary file 2 [file mmc2.zip › mmc2.tif]

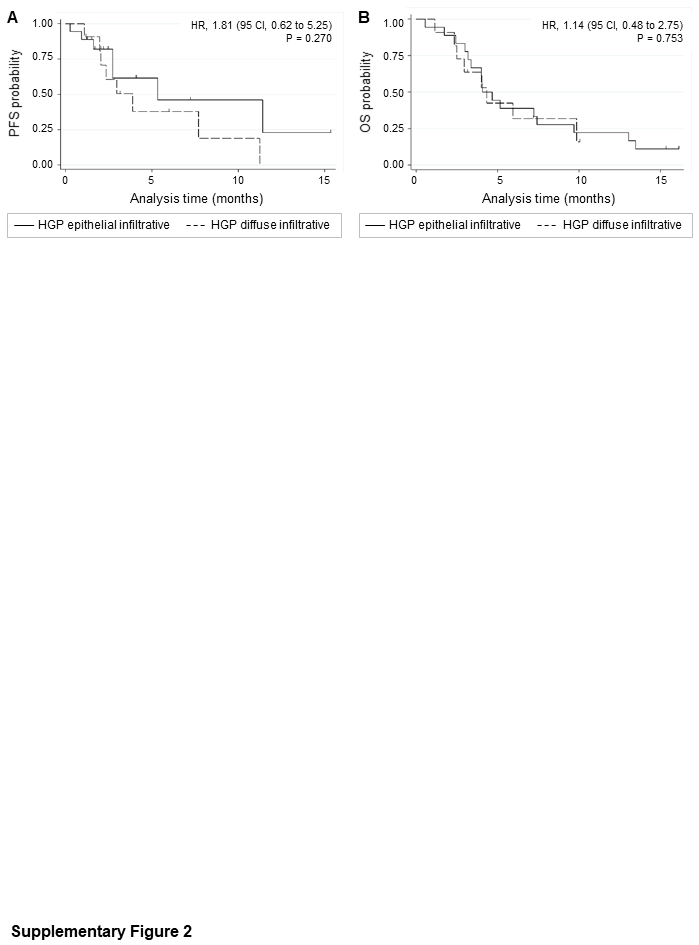

Supplement: Supplementary file 3 [file mmc3.zip › mmc3.tif]
